# Interfacial polygonal patterning via surfactant-mediated self-assembly of gold nanoparticles

**DOI:** 10.1186/1556-276X-8-436

**Published:** 2013-10-22

**Authors:** Yu Xin Zhang, Xiao Dong Hao, Min Kuang, Ru De Chen

**Affiliations:** 1 College of Materials Science and Engineering, Chongqing University, Chongqing 400044, People’s Republic of China; 2 National Key Laboratory of Fundamental Science of Micro/Nano-Devices and System Technology, Chongqing University, Chongqing 400044, People’s Republic of China; 3 Department of Chemical and Biomolecular Engineering, Faculty of Engineering, National University of Singapore, 10 Kent Ridge Crescent, Singapore 119260, Singapore

**Keywords:** Nanoparticles, Interfaces, Thin films, Hybrid, Self-assembly, Polygonal pattern

## Abstract

In this work, we explored the formation processes of interfacial polygonal patterning via surfactant-mediated self-assembly of gold nanoparticles (AuNPs). We found that a balance between DDT-capped AuNPs and PVP-passivated AuNPs is a key to making these inorganic–organic thin films. The interfacial polygonal patterning possesses many processing advantages and flexibilities, such as controllable interfacial shape and inter-AuNP distance, tuning of particle sizes, thiol population, chain lengths, and other new properties by introducing functional groups to thiol chains. In principle, self-assembly of AuNPs via well-designed interfaces may be useful for fabrications of other complex architectures.

## Background

Gold nanoparticles (AuNPs) are among the most studied nanomaterials in recent years, owing to their outstanding properties in catalytic, electrical, optical, and biomedical applications [1-9]. The controlled fabrication of gold nanoparticles at scales beyond the current limits of characterization techniques is a technological goal of practical and fundamental interest. Important progress has been made over the past few years in the self-assembly and organization of Au nanostructures ranging from one-, two-, and three-dimensional (1D, 2D, and 3D) ordered arrays and superlattices to random aggregates and superstructures [1-14]. While most of this research endeavor relies on the van der Waals interaction of surfactants adsorbed on the surfaces of AuNPs, several types of constructional assemblies have been well developed, in which as-synthesized AuNPs were used as primary building units to grow larger monodisperse particles and to construct continuous 3D networks under heat conditions. One important area remaining to be explored is whether these preassembled AuNPs can be used as structure precursors for fabricating other even more complex Au nanostructures when surface organics are controllably removed [15-25]. Herein, we devise a new synthetic protocol, which combines both surfactant-assisted assembly and heat-activated attachment, to generate interfacial polygonal patterning of self-assembled nanostructures [15]. In particular, we will use small AuNPs (2 to 5 nm in size) as starting units to fabricate several different kinds of complex gold nanostructures in polygonal patterning with a high morphological yield of 100%.

## Methods

### Synthesis of interfacial polygonal patterning via self-assembly of Au nanoparticles

Thiol-capped Au seeds were prepared by Brust's two-phase method with some minor modifications (see Additional file 1 for the detailed synthesis procedure) [11,16,21,22]. In a typical experiment, two standard units (denoted as STUs) of Au nanoparticles were redissolved in cyclohexane (2 mL for each STU), followed by the addition of PVP (1.25 mM, 0.5 mL in 2-propanol) and DDT (0.11 M, 22 mL in cyclohexane). The obtained mixture was then placed into a Teflon-lined stainless steel autoclave, and the solvothermal synthesis was conducted at 150°C to 210°C for 2 to 14 h in an electric oven. After the reactions, gold products were harvested by centrifuging and dissolved into ethanol solvent for their stabilization. Detailed preparative parameters adopted in the above experiments can be found in Additional file 1: SI-1. The as-prepared gold nanomaterial products were characterized with transmission electron microscopy (TEM; JEM2010F, JEOL Ltd., Akishima-shi, Tokyo, Japan) and field-emission scanning electron microscopy (FESEM; JSM-6700F, JEOL Ltd., Akishima-shi).

## Results and discussion

**Figure 1:**
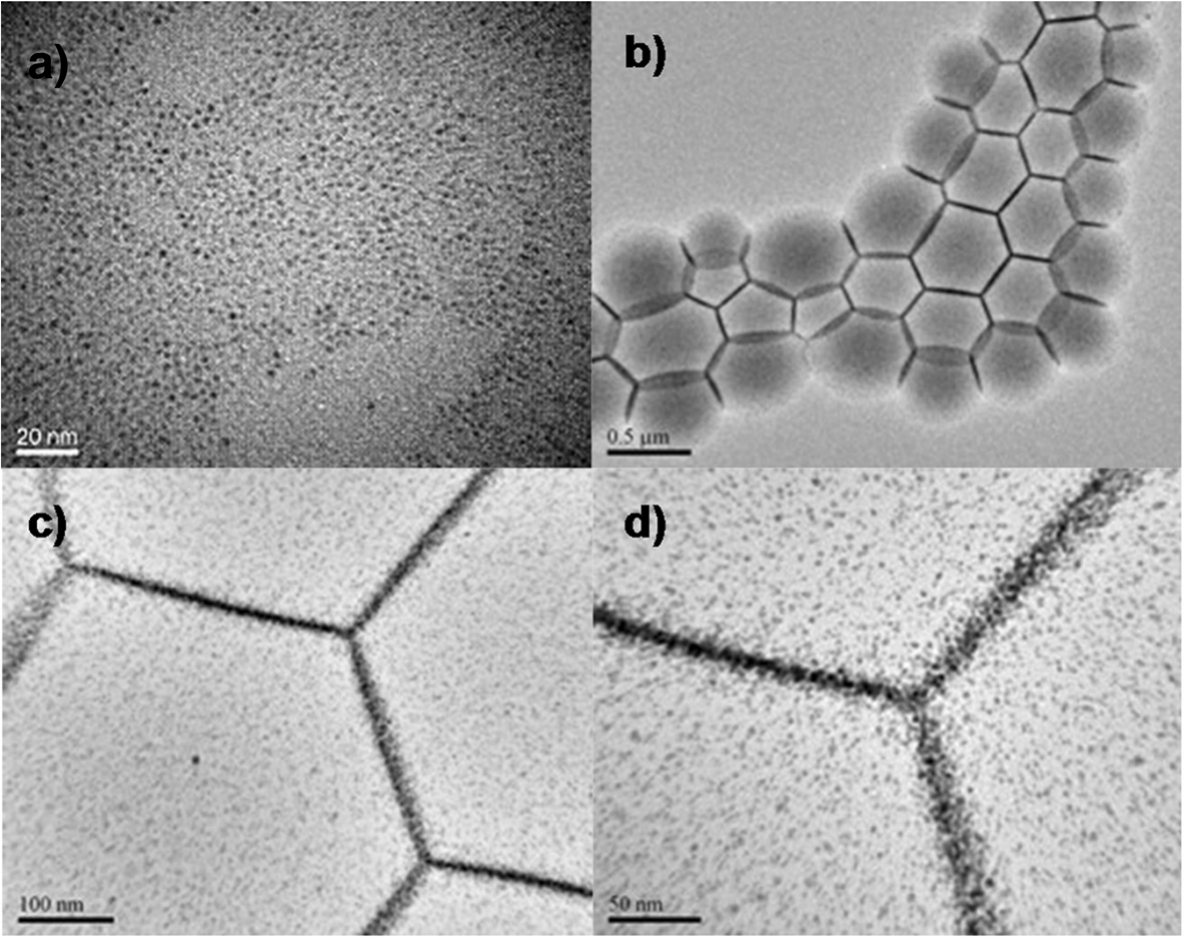
**TEM images. (a)** Typical Au nanoparticles as building units and **(b** to **d)** typical interfacial polygonal patterning via surfactant-mediated self-assembly of Au nanoparticles with different magnifications. Experimental conditions: AuNPs (Au/DDT = 0.1); AuNPs (2STU) + DDT (0.11 M; 22 mL) + PVP (1.25 mM; 0.5 mL), 180°C, 4 h. See Additional file 1: SI-1 for more information on their detailed experimental conditions.

Figure 1a shows an example of Au nanoparticles (2 to 3 nm) packed in hexagonal organization. As building units, AuNPs are organized into interfacial polygonal patterning for the first time, exhibiting a remarkable degree of long-range order. Intriguingly, a distribution of hexagon, pentagon, and complex patterns can be clearly observed (Figure 1b), which had typical lateral dimensions such as scale approximately 500 nm. (Isolated bubbles with radii mostly greater than 300 nm were stable over a period of a few months)Under high magnification (Figure 1c,d), it is more clear that AuNPs are assembled into solid laterals (e.g., thickness 5 to 20 nm) with higher concentrations of AuNP aggregations, while loose dispersed AuNPs are distributed within polygonal patterning. Surprisingly, the internal angles approximately equal to 120° (120° ±1°). Although the resultant patterns resemble the nanopatterning of stable microbubbles [15], herein the patternings of self-assembly of gold nanoparticles can be more potentially functional and stabilized. Inasmuch, improvements in stabilization could be achieved by coating the interface with nanoparticles.

**Figure 2:**
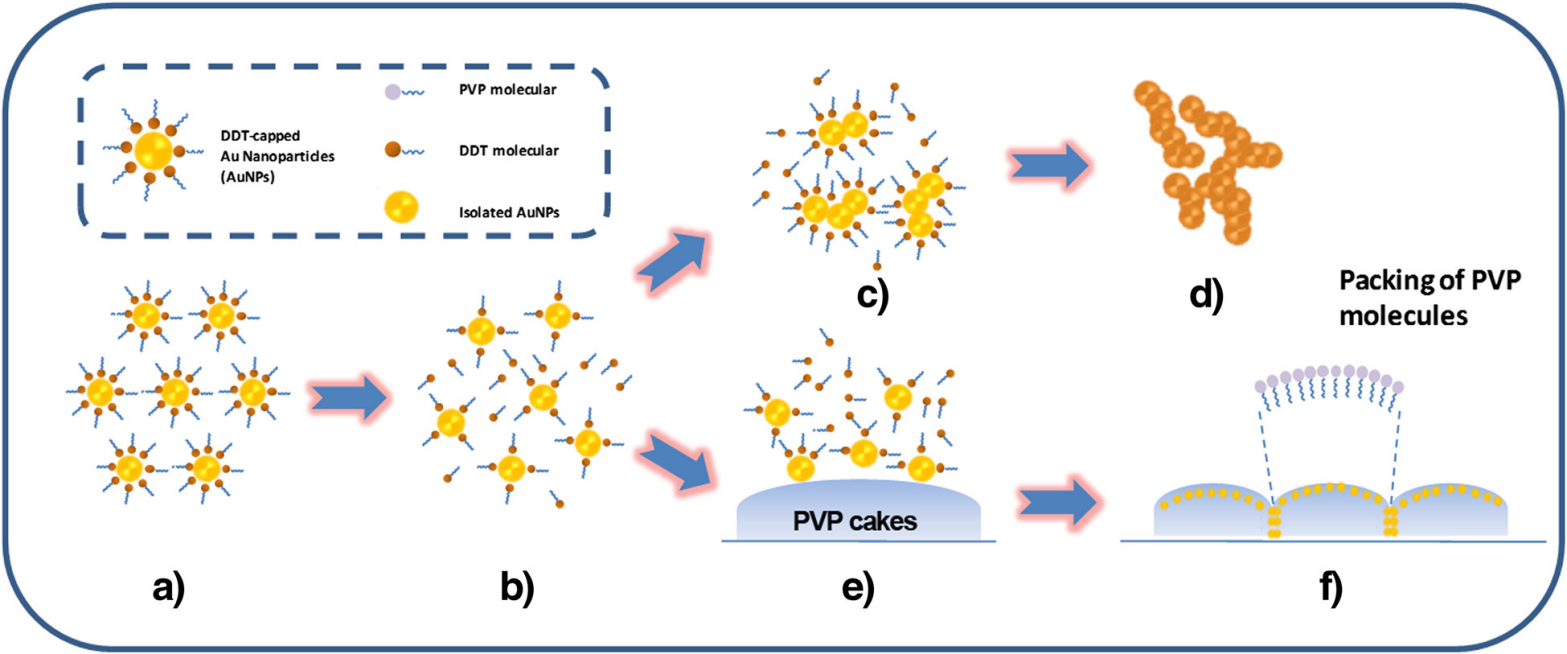
**Schematic illustrations.** Formation of interfacial polygonal patterning via **(a** to **b** and **b** to **f)** surfactant-mediated self-assembly of gold nanoparticles and **(a** to **b** and **b** to **d)** formation of gold sponges. The insets stand for the figure legend.

To further uncover the interfacial polygonal patterning, Figure 2 depicts formation route using functionalized AuNPs. Under ambient conditions, DDT-capped AuNPs tend to agglomerate together via van der Waals forces generated among their surface alkyl headgroups (Figure 2a). Upon heating, it is clear that solvothermal process is an essential condition to activate surface reactivity of AuNPs (Figure 2b) and thus to initiate 3D networking (Figure 2c,d), though alcohol washing (hydrothermal washing) may also facilitate this process. On the other hand, chemical conversion of DDT to sulfate salt under the same hydrothermal condition can also reduce total amount of DDT in the synthetic system, which is equivalent to the partial removal of DDT surfactant. Since it is still adsorbed on the Au sponges (Figure 2d) after particle aggregation, the DDT also serves as a protecting reagent for the product. Therefore, the key to the formation of sponges lies on the manipulation of alkanethiol content in the synthesis. Additionally, PVP, as one of ideal candidates of surfactants for gold nanostructures [12], is implemented to fabricate functional interfaces by virtue of its variant solubility in different solvents (i.e., cyclohexane and 2-propanol). Ideally, patterned PVP cakes were built at the bottom of Teflon liner, exhibiting their flat planes with spherical-cap appearance (Figure 2e). The interfacial structures (Figure 2f) were depicted, resulting from the packing of PVP molecules. As a further confirmation, detached or naked AuNPs were captured by tentacles and embedded into PVP cakes due to the affinity of Au and PVP. Thereupon, the formation process presents binary assembly, including PVP cakes assembly (i.e., interface fabrication) and assembly of AuNPs on PVP cakes, inorganic–organic nanocomposites in nature.

With respect to detailed investigation, two types of patterns such as hexagonal or complex patterns were proposed combined with patterns of foamed construction materials. Indeed, the bubbles continuously evolve toward lower-energy configuration by minimizing the interfacial area, so that we could obviously observe the spherical outlines along PVP cakes standing aside. The PVP cakes inside could compress the surrounding cakes to pursue an equilibrium of interfacial tension, which lies in the size of PVP cakes, exhibiting a perpendicular plane among the cakes. More quantitatively, solid laterals or arc laterals among the patterning could be observed from top and side view. Due to lack of adequate surrounding cakes, the cakes outside could penetrate into the bottom of the ones inside, exhibiting an arc lateral from side view, and/or two crossed arcs from top view.

**Figure 3:**
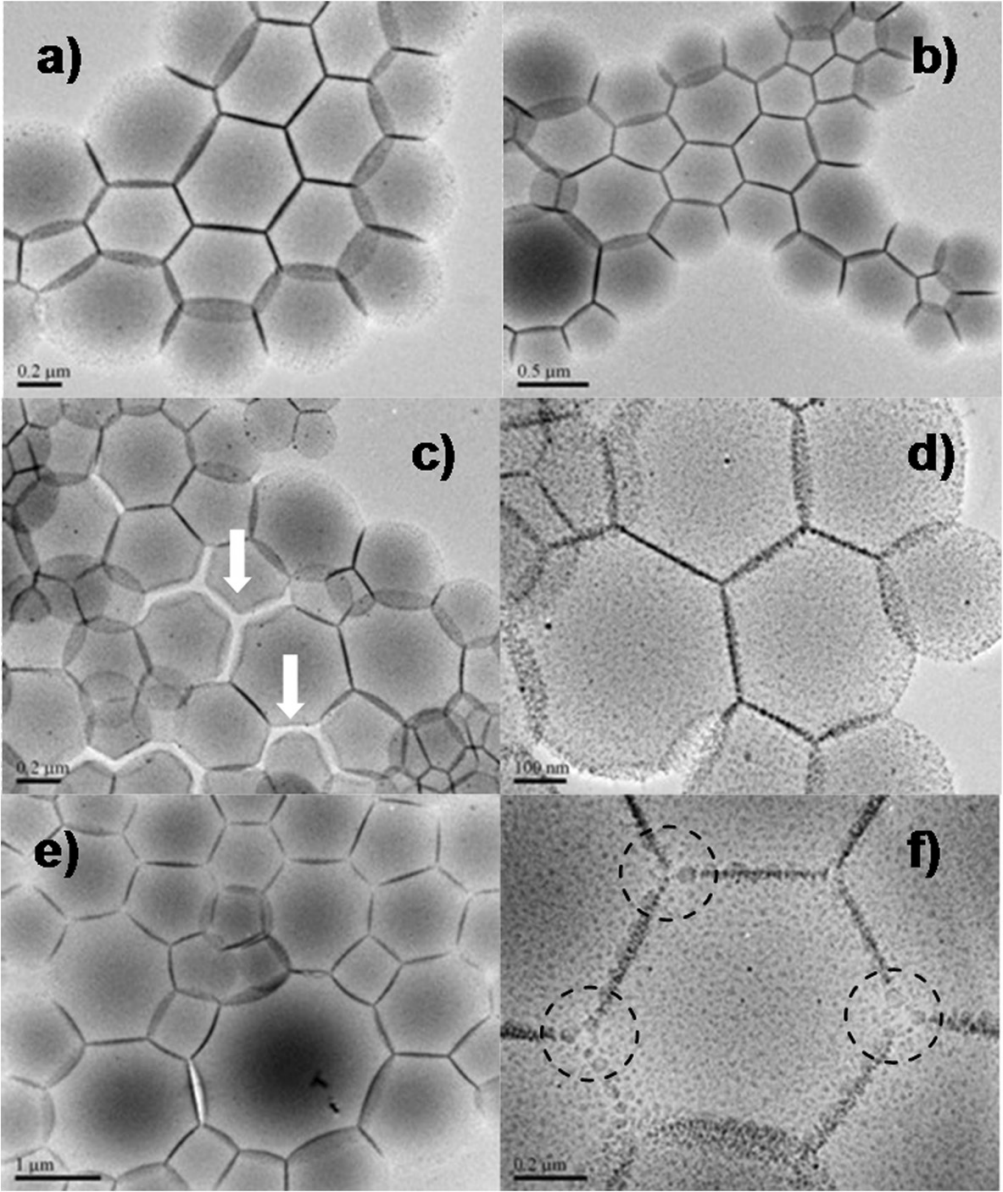
**TEM images.** Typical interfacial polygonal patterning - experimental conditions: AuNPs (2STU) + DDT (0.11 M) + PVP (1.25 mM), 180°C, 4 h. **(a, b)** Au/DDT = 0.1, DDT (22 mL); **(c, d)** Au/DDT = 0.2, DDT (22 mL); **(e, f)** Au/DDT = 0.1, DDT (22 mL); See Additional file 1: SI-1 for more information on their detailed experimental conditions.

On the basis of our previous studies [11,22], interfacial polygonal patterning could be tuned by manipulating surfactant population, concentration of metallic nanoparticles, amount and type of PVP in 2-propanol, process temperature and time, etc. Herein, the surfactant population is manipulated with modified modes at different stages: synthesis of AuNPs (pristine anchored DDTs) and solvothermal treatment of AuNPs (freshly supplementary DDTs). For instance, Au seeds (Au/DDT=0.1) was mixed with freshly prepared DDT (0.11 M, 22 mL) and PVP (1.25 mM, 0.5 mL), followed by solvothermal treatment (180°C and 4 h). The resultant products are presented in Figure 3a,b, exhibiting apparent and close-packed interfacial polygonal patterning. When anchored DDT on Au seeds is decreased, the voids (pointed out by white arrow in Figure 3c) appear to form loose-packed cakes. Under identical conditions, 2 mL of fresh DDT (isolated DDT molecules, Figure 2b) was added in, leading to charcoal-drawing patterning with snatch laterals. Surprisingly, in the interconnection zones among three cakes are very sparsely distributed AuNPs, pointed out by dotted circle (Figure 3f). Very few voids also could be observed in Figure 3e. As noted earlier, the generation of interior porosity is apparently associated with the depletion of anchored surfactants and direct attachment among the AuNPs.

To further confirm the synergistic effect of PVP and DDT, the effects of stand-alone surfactant-mediated self-assembled nanostructures are carried out first (see Additional file 1: SI-2). Besides PVP in-2 propanol solvent (without any addition of fresh DDT), solid PVP powders were also used to tailor self-assembly of AuNPs. Meanwhile, various amounts of freshly prepared DDT were applied to fine tune the gold nanostructures. Nevertheless, the morphology yields for resultant products as gold sponges are extremely high at about 100% instead of interfacial polygonal patterning. If the process time is short enough, or freshly prepared DDT are abundantly added, the worm-like gold nanostructures or closed-packed hexagonal AuNPs (after growth) were obtained, respectively. In light of the above findings, our time-dependent synthesis with combined surfactants was executed to make clear real roles of the surfactants alone. As shown in Additional file 1: SI-3a, the contour outlines of PVP cakes with gold nanoparticles were clearly explored, followed by interlinks of PVP cakes (Additional file 1: SI-3c) and AuNPs aggregates (Additional file 1: SI-3d) on the cakes. Finally, the mixture of soft PVP assemblies and Au sponges was harvested after 5-h heat treatment (Additional file 1: SI-3e,f). On the basis of systematical studies, the optimal process time and temperature can be ruled out as 4 h and 180°C. Particularly, from the Additional file 1: SI-3, it also proved that higher concentration of PVP in 2-propanol (5 mM, 0.5 mL) went against the formation of interfacial polygonal patterning.

**Figure 4:**
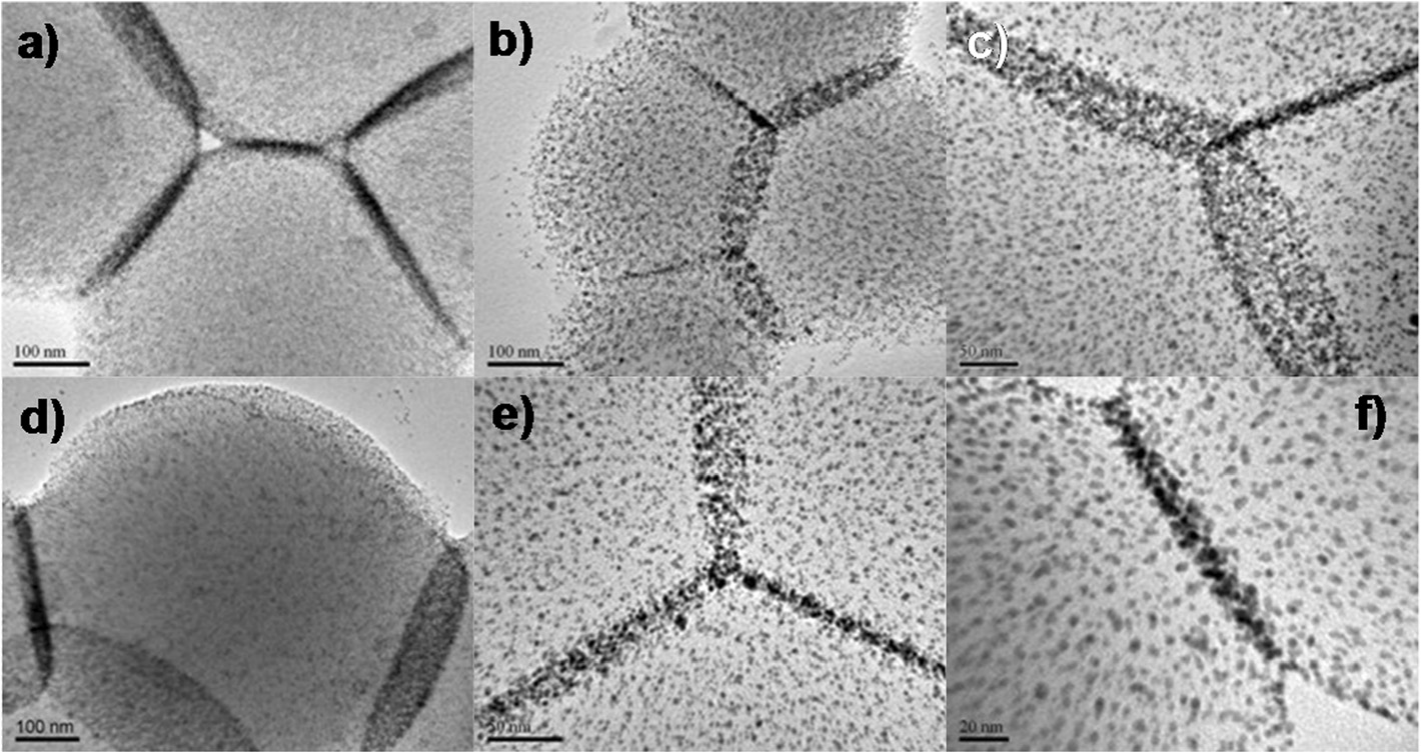
**TEM images.** Various kinds of borders in interfacial polygonal patterning-experimental conditions: AuNPs (2STU) + DDT (0.11 M) + PVP (1.25 mM), 180°C, 4 h. **(a)** Au/DDT = 1, DDT (22 mL); **(b)** Au/DDT = 1, DDT (4 mL); **(c)** Au/DDT = 1, DDT (2 mL), PVP (5 mM, 0.5 mL); **(d)** Au/DDT = 1, DDT (2 mL); **(e)** Au/DDT = 0.1, DDT (22 mL); **(f)** Au/DDT = 1 and Au/DDT = 0.2, DDT (2 mL); See Additional file 1: SI-1 for more information on their detailed experimental conditions.

It is understandable that these surfactants must be well manipulated if an evolution of interfacial polygonal patterning is achieved. In relation to the structural tailoring, the surfactants (DDT) must be partially removed if a crystal growth or coupling is engaged. And thus, 2-propanol solvent has been proved to be efficient for the surfactant removal within reasonable dosage corresponding to cyclohexane under solvothermal conditions. As noted earlier in Figure 2, by selecting a set of preparative parameters, for example, various kinds of borders in interfacial polygonal patterning have been made (Figure 4): arc laterals (Figure 4a,b,c,d), solid line laterals (Figure 4f), and mixed laterals (Figure 4e). It should be announced that assembled nanostructures seem like cakes rather than the spheres, judged by virtue of the curved edges (Figures 4b and 3d). Unlike popular core-shell structures, interfacial polygonal patterning did not own their pronounced shell, assembled with nanoparticles. FESEM images in Additional file 1: SI-4 also prove the truth of the nature of soft cakes regarding to interfacial polygonal patterning. As a result of assemblies of cakes, the solid or curved lines in TEM images were composed of the project of nanoparticles with different heights, embedded in the surface of PVP cakes. The area of project planes is determined by sizes of cakes and their surrounding conditions. And thus, the solid or arc laterals could be observed in Figure 4, indicating two primary types of interfacial polygonal patternings.

**Figure 5:**
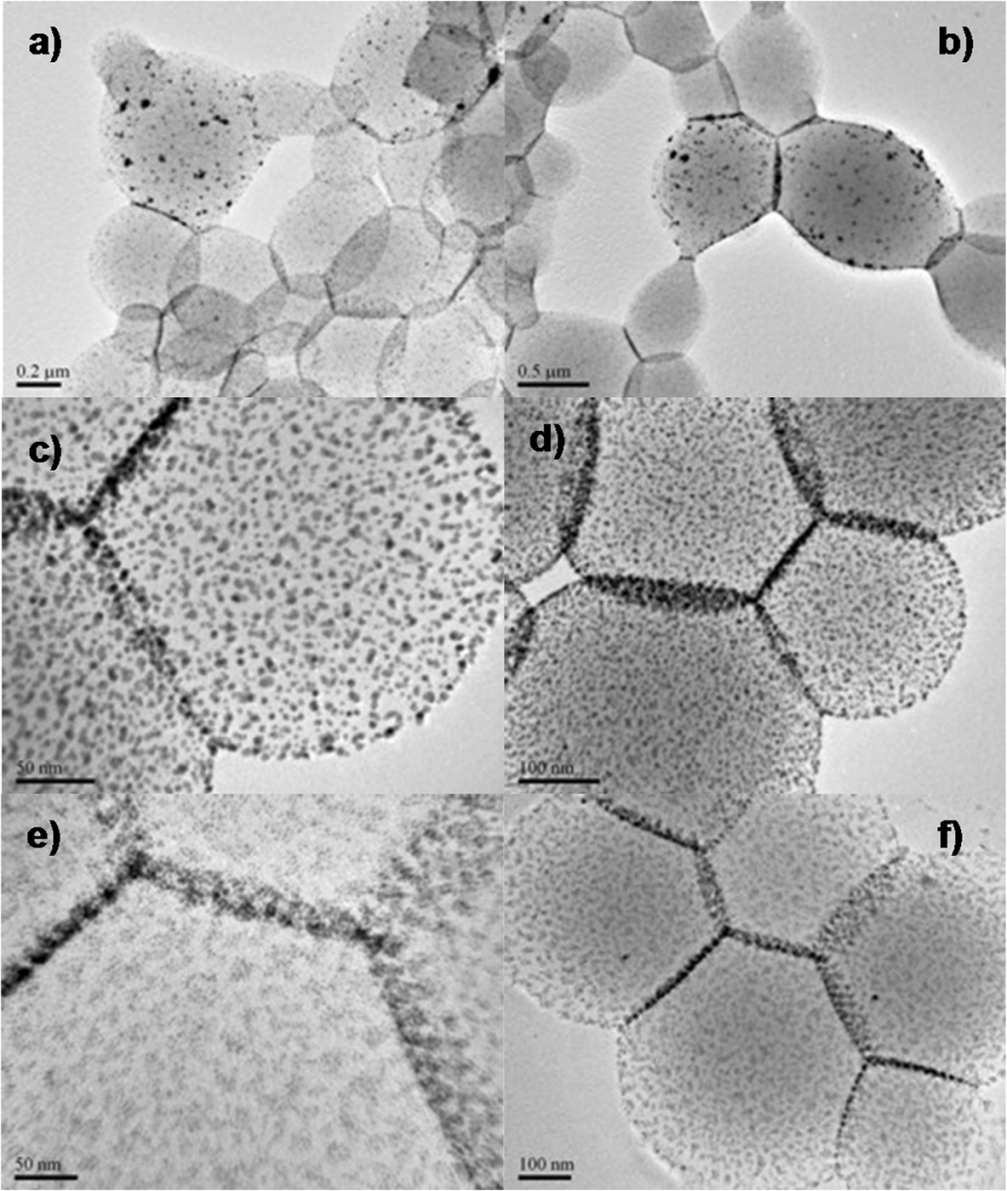
**TEM images.** Functional interfacial polygonal patterning with mixing various Au seeds - experimental conditions: AuNPs (2STU) + DDT (0.11 M) + PVP (1.25 mM), 180°C, 4 h. **(a, b)** Au/DDT = 10 and Au/DDT = 0.02, DDT (2 mL); **(c, d)** Au/DDT = 5 and Au/DDT = 0.02, DDT (2 mL); **(e, f)** Au/DDT = 0.2 and Au/DDT = 0.1, DDT (2 mL); See Additional file 1: SI-1 for more information on their detailed experimental conditions.

In order to explore the functionality of interfacial polygonal patternings, there are several preparative parameters, such as concentration of gold nanoparticles precursors and combinations of binary AuNPs, manipulated to fine tune the interparticle distances or binary nanoparticle assemblies. Figure 5 presents the typical functional interfacial polygonal patterning with mixing various Au seeds. Figure 5a,b shows an example of interfacial polygonal patterning where particles of 2 to 3 nm and 10 to 13 nm in diameter are packed in dispersed manner, exhibiting a remarkable degree of tunable particle size distribution. Here, as in all other cases (Figure 5c,d,e,f), adjacent AuNPs were separated by different distances, which is considerably adjustable by the expected thiol chain length and PVP molecules. In principle, functionalities of interfacial polygonal patternings enable these films useful for biosensor or catalysis applications.

## Conclusions

In summary, for the first time, we have developed a self-assembly approach for generation of interfacial polygonal patterning with as-synthesized AuNPs as starting building blocks. It is found that the hydrothermal condition is essential to detach DDT and PVP surfactants and thus trigger the self-assembly of AuNPs. The resultant interfacial polygonal patterning can be further controlled by manipulating surfactant morphology, concentration of metallic nanoparticles, amount of surfactants, process temperature and time, etc. In principle, this self-assembly approach can also be extended to large-scale 3D organizations of other surfactant-capped transition/noble metal nanoparticles.

## Abbreviations

AuNPs: Au nanoparticles; DDT: 1-dodecanethiol; STU: Standard unit; TEM: Transmission electron microscopy; FESEM: Field-emission scanning electron microscopy; SI: Supporting information.

## Competing interests

The authors declare that they have no competing interests.

## Authors’ contributions

ZYX synthesized the self-assembled samples and wrote the manuscript. HXD, KM, and CRD characterized the self-assembled samples and coordinated the experiments. All authors read and approved the final manuscript.

## Supplementary Material

Additional file 1 Includes six Additional files (SI): (1) Synthetic conditions; (2) TEM images of stand-alone surfactants mediated self-assembly nanostructures; (3) TEM images of combined surfactants mediated self-assembly nanostructures under different processing time at 180°C; (4) FESEM images of combined surfactants mediated self-assembly nanostructures at 180°C for 4 h; (5) TEM images of combined surfactants mediated self-assembly nanostructures at 180°C for 4 h; (6) TEM images of typical interfacial polygonal patterning. Click here for file
