# Supplementary material for: Interfacial polygonal patterning via surfactant-mediated self-assembly of gold nanoparticles
Source: Nanoscale Res Lett. 2013 Oct 22;8(1):436. doi: 10.1186/1556-276X-8-436 (PMC4015365; doi:10.1186/1556-276X-8-436)
Supplement: Additional file 1 — Includes six Additional files (SI): (1) Synthetic conditions; (2) TEM images of stand-alone surfactants mediated self-assembly nanostructures; (3) TEM images of combined surfactants mediated self-assembly nanostructures under different processing time at 180°C; (4) FESEM images of combined surfactants mediated self-assembly nanostructures at 180°C for 4 h; (5) TEM images of combined surfactants mediated self-assembly nanostructures at 180°C for 4 h; (6) TEM images of typical interfacial polygonal patterning. [file 1556-276X-8-436-S1.docx]

**Interfacial Polygonal Patterning via Surfactant-mediated Self-assembly of Gold Nanoparticles**

Yu Xin Zhang ^1,2*^ , Xiao Dong Hao ^1^, Min Kuang^1^ , Ru De Chen^2^

*Corresponding Author

Email: zhangyuxin@cqu.edu.cn

^1^ College of Materials Science and Engineering, Chongqing University, Chongqing, 400044, P.R.China

^2^ National Key Laboratory of Fundamental Science of Micro/Nano-Devices and System Technology, Chongqing University, Chongqing, 400044, P.R. China

^3^ Department of Chemical and Biomolecular Engineering, Faculty of Engineering, National University of Singapore, 10 Kent Ridge Crescent, Singapore, 119260, Singapore

**Additional file**

**SI-1 Synthetic conditions**

1. **Synthesis of Au nanoparticles.** Thiol-capped Au seeds were prepared by Brust’s two-phase method with some minor modifications. Briefly, a hydrogen tetrachloroaurate trihydrate (HAuCl_4_·3H_2_O, at 30 mM) aqueous solution (3.0 mL) was added to a tetraoctylammonium bromide (TOAB, at 50 mM), containing toluene solution (3.6 mL), and resultant solution was thoroughly mixed, during which the aqueous phase turned from yellow to colorless while the organic phase turned orange as a result of the transformation of [AuCl_4_]^-^ with TOAB cations. Under the stirring condition at room temperature, the solution was further mixed with 1-dodecanethiol (DDT, 0.11 M in toluene, 0.82 mL) for 15 min, followed by adding a freshly prepared sodium borohydride (NaBH_4_, at 0.44 M in deionized water, 2.05 mL) solution. The above mixture immediately turned from orange to deep brown, and the resulting suspension was continuously stirred for another 15 min. Finally, the organic phase (~4.5 mL) was collected and uniformly divided into eight units (sample vials). In each standard unit (denoted as STU), isolation of the derivatized cluster was achieved by precipitation following addition of methanol, and the pure solid product was obtained after centrifugation and spontaneous drying process, to remove excessive of reagents and solvents. Unless otherwise specified, in most syntheses of this work the Au nanoparticles were prepared at Au/TOAB=0.5, Au/DDT=1, and Au/NaBH_4_=0.1 according to the above procedure. It is noted that the proportion of Au:DDT used in the synthesis controls the size of Au nanoparticles. In our synthesis, the ratio of Au and DDT included 0.02, 0.1, 0.2, 1, 5, and 10 to pursue different size of Au nanoparticles.
2. **Standard Units of AuNPs (STUs)**: Schematic illustration of sample washing.

1. **Detailed structures of surfactants (DDT and PVP)**.


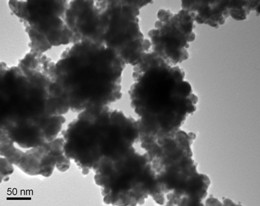

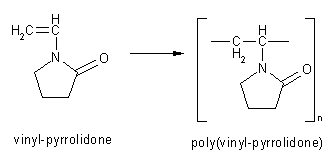

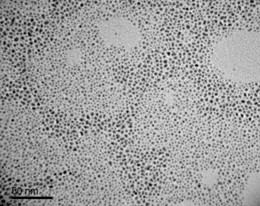

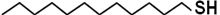


**DDT**: to inhibit the Ostwald ripening;

**PVP**: to accelerate the Ostwald ripening;

1. Polygonal patterns from real life


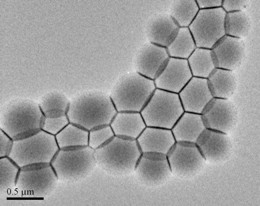

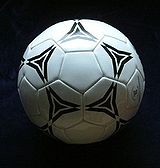

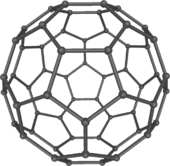


**(c)**

**(b)**

**(a)**

1. AuNPs self-assembled pattern; (b) Fullerene (from Wikipedia); (c) Fussball(from Wikipedia)
2. **Detailed experimental conditions for all the samples**.

| **No. of Samples** | **Au/DDT**  **(ratio)** | **STU** | **DDT**  **(M/mL)** | **PVP**  **(M/mL)** | **Temperature**  **(^o^C)** | **Time**  **(hours)** | **Figures** |
| --- | --- | --- | --- | --- | --- | --- | --- |
| 1 | 0.1 | - | - | - | - | - | Fig.1a |
| 2 | 0.1 | 2 | 0.11/22 | 0.00125/0.5 | 180 | 4 | Fig.1b-d  Fig.3a-b  Fig.4e  SI.4 |
| 3 | 0.2 | 2 | 0.11/22 | 0.00125/0.5 | 180 | 4 | Fig.3c-d |
| 4 | 0.1 | 2 | 0.11/2 | 0.00125/0.5 | 180 | 4 | Fig.3e-f |
| 5 | 1 | 2 | 0.11/22 | 0.00125/0.5 | 180 | 4 | Fig.4a  SI.5e-f |
| 6 | 1 | 2 | 0.11/4 | 0.00125/0.5 | 180 | 4 | Fig.4b  SI.5c-d |
| 7 | 1 | 2 | 0.11/2 | 0.005/0.5 | 180 | 4 | Fig.4c  SI.3c-d  SI.5g-h |
| 8 | 1 | 2 | 0.11/2 | 0.00125/0.5 | 180 | 4 | Fig.4d  SI.5a-b |
| 9 | 1/0.2 | 1/1 | 0.11/2 | 0.00125/0.5 | 180 | 4 | Fig.4f |
| 10 | 10/0.02 | 1/1 | 0.11/2 | 0.00125/0.5 | 180 | 4 | Fig.5a-b |
| 11 | 5/0.02 | 1/1 | 0.11/2 | 0.00125/0.5 | 180 | 4 | Fig.5c-d |
| 12 | 0.2/0.1 | 1/1 | 0.11/2 | 0.00125/0.5 | 180 | 4 | Fig.5e-f |
| 13 | 1 | 2 | - | 0.1g | 180 | 4 | SI.2a |
| 14 | 1 | 2 | - | 0.00125/0.5 | 180 | 2 | SI.2b |
| 15 | 1 | 2 | - | 0.00125/0.5 | 180 | 3 | SI.2c |
| 16 | 1 | 2 | - | 0.00125/0.5 | 180 | 4 | SI.2d |
| 17 | 1 | 2 | 0.11/2 | - | 180 | 5 | SI.2e |
| 18 | 1 | 2 | 0.11/4 | - | 180 | 4 | SI.2f |
| 19 | 1 | 2 | 0.11/6 | - | 180 | 4 | SI.2g |
| 20 | 1 | 2 | 0.11/22 | - | 180 | 4 | SI.2h |
| 21 | 1 | 2 | 0.11/2 | 0.05/0.5 | 180 | 3 | SI. 3a-b |
| 22 | 1 | 2 | 0.11/2 | 0.05/0.5 | 180 | 5 | SI. 3e-f |
| 23 | 0.2 | 2 | 0.11/2 | 0.00125/0.5 | 180 | 4 | SI.6a-b |
| 24 | 0.02 | 2 | 0.11/2 | 0.00125/0.5 | 180 | 4 | SI.6c-d |
| 25 | 5 | 2 | 0.11/2 | 0.00125/0.5 | 180 | 4 | SI.6e-f |

Note: One STU denotes one bottle of as-obtained gold nanoparticle after drying.

**SI-2** TEM images of stand-alone surfactants mediated self-assembly nanostructures: (a) solid PVP powder (0.1 g) at 180 ^o^C for 4 h; PVP in 2-propanol (1.25 mM, 0.5 mL) at 180 ^o^C for different processing time: (b) 2 h; (c) 3 h; (d) 4 h; Different DDT addition (0.11 M) at 180 ^o^C for 4 h: (e) 2 mL; (f) 4 mL;(g) 6 mL;(h) 22 mL. See Additonal File (SI-1) for more information on their detailed experimental conditions.

**d)**


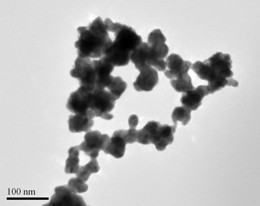

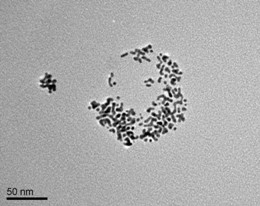

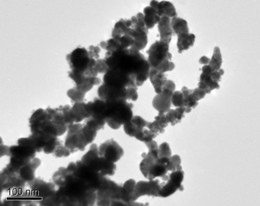

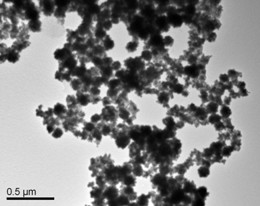


**a)**

**b)**

**c)**


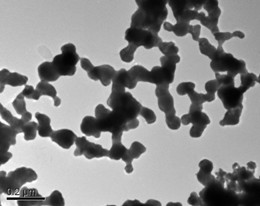

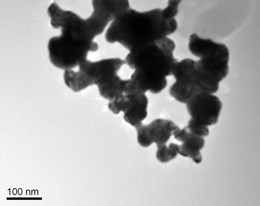

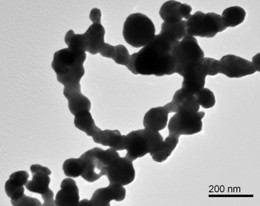

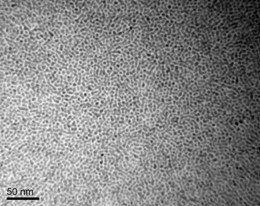


**e)**

**f)**

**g)**

**h)**

**SI-3** TEM images of combined surfactants mediated self-assembly nanostructures under different processing time at 180 ^o^C: (a-b) 3 h; (c-d) 4 h; (e-f) 5 h. The inset of (f) is high magnification of area in dotted rectangle. See Additonal File (SI-1) for more information on their detailed experimental conditions.


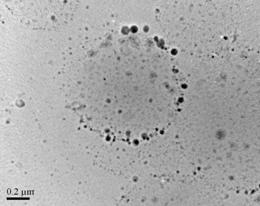

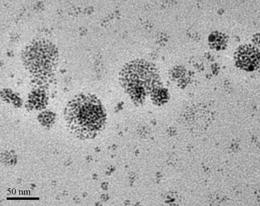

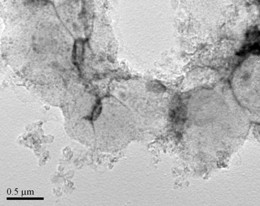

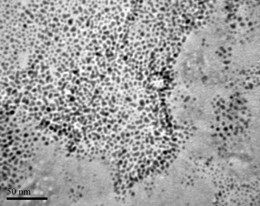

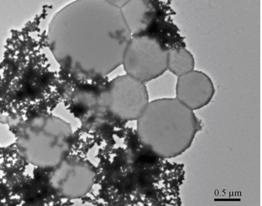

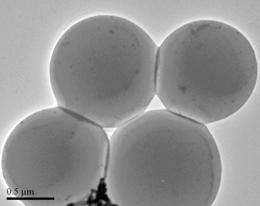

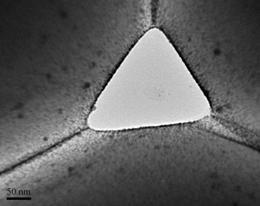


**a)**

**b)**

**c)**

**d)**

**e)**

**f)**

**SI-4** FESEM images of combined surfactants mediated self-assembly nanostructures at 180 ^o^C for 4 h. See Additonal File (SI-1) for more information on their detailed experimental conditions.


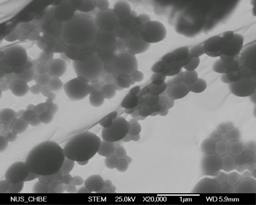


**b)**


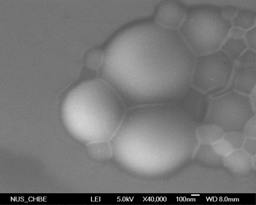


**a)**

**SI-5.** TEM images of combined surfactants mediated self-assembly nanostructures at 180 ^o^C for 4 h. (a-b) 0.5 mL of PVP (1.25 mM@2-propanol) + 2 mL of DDT (0.11 M@ cyclohexane); (c-d) 0.5 mL of PVP (1.25 mM@2-propanol) + 4 mL of DDT (0.11 M@ cyclohexane); (e-f) 0.5 mL of PVP (1.25 mM@2-propanol) + 22 mL of DDT (0.11 M@ cyclohexane); (g-h) 0.5 mL of PVP (5 mM@2-propanol) + 2 mL of DDT (0.11 M@ cyclohexane). See Additonal File (SI-1) for more information on their detailed experimental conditions.


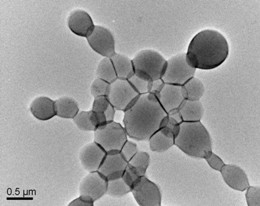

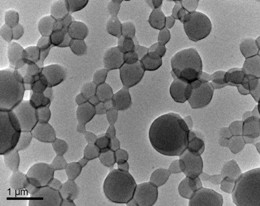

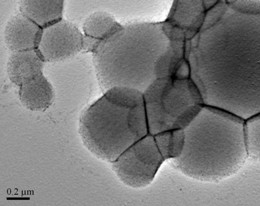

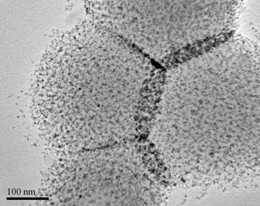

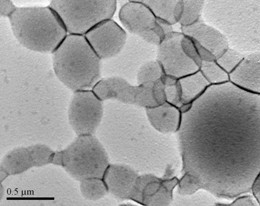

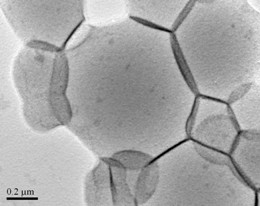

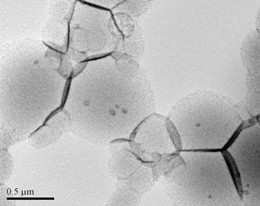

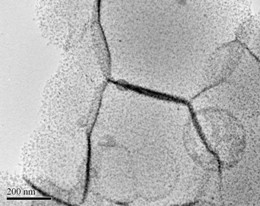


**a)**

**b)**

**c)**

**e)**

**f)**

**g)**

**h)**

**d)**

**SI-6** TEM images of typical interfacial polygonal patterning. See Additonal File (SI-1) for more information on their detailed experimental conditions.


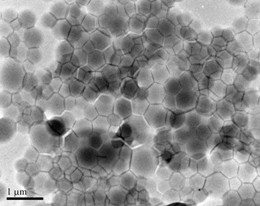

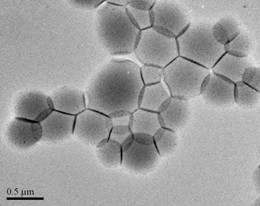

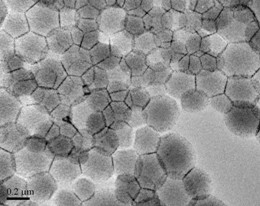

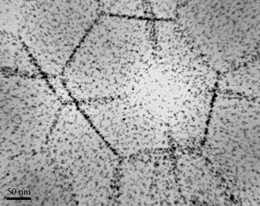

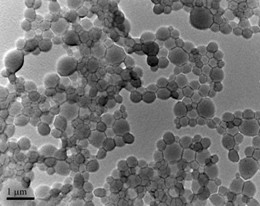

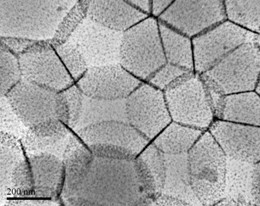


**a)**

**b)**

**c)**

**e)**

**f)**

**d)**
